# Shared Decision Making in Acute Pain Management in Patients with Opioid Use Disorder: A Scoping Review

**DOI:** 10.3390/jcm12103555

**Published:** 2023-05-19

**Authors:** Peter D. Vu, Aila Malik, A. Sarah Cohen, Vishal Bansal, Morgan R. Cowan, Gregory M. Blazek, Tiffany Champagne-Langabeer

**Affiliations:** 1 Department of Physical Medicine and Rehabilitation, McGovern Medical School, UTHealth Houston, 6431 Fannin, Houston, TX 77030, USA; 2 Houston ER Opioid System (HEROES), School of Biomedical Informatics, UTHealth Houston, 7000 Fannin, Houston, TX 77030, USA; 3 Tiffany Champagne-Langabeer, UTHealth Houston, 7000 Fannin St., Suite 600, Houston, TX 77030, USA

**Keywords:** opioid use disorder, acute pain, pain management, shared decision making, scoping review

## Abstract

The treatment of acute pain over the years has changed with increasing alternative therapies and increased scrutiny of opioid prescriptions. Shared Decision Making (SDM) has become a vital tool in increasing patient engagement and satisfaction in treatment decisions. SDM has been successfully implemented in the management of pain in a variety of settings; however, information regarding the use of SDM for treating acute pain in patients with a history of opioid use disorder (OUD) remains scarce. Following the Preferred Reporting Items for Systematic Reviews and Meta-analysis Extension for Scoping Reviews (PRISMA-ScR), we conducted a review to understand how SDM is used in acute pain management in patients with OUD. We searched Medline, Embase, CINAHL, and PsychInfo databases for relevant articles. Articles were screened and SDM outcomes of eligible articles were charted. The results were grouped by sub-theme based on a 1997 SDM model. There were three original research studies and one quality improvement study. The remaining articles were split evenly between reviews and reviews of clinical guidelines. Four themes emerged from the review: prior judgment and stigma related to OUD, trust and sharing of information, clinical tools, and interprofessional teams. This scoping review consolidated and expounded the current literature on SDM in the management of acute pain in patients with OUD. More work is needed to address prior judgments by both providers and patients and to build greater dialogue. Clinical tools may aid this process as well as the involvement of a multidisciplinary team.

## 1. Introduction

Since the 1987 World Health Organization (WHO) analgesic ladder was first published, knowledge of pain physiology and the clinical management of pain has evolved [1]. In an effort to improve the treatment of cancer pain, this step-wise approach ranged from the use of non-opioid analgesics for mild pain to strong opioid agents for moderate to severe cases. Over the years, the ladder has undergone several updates by various authorities and has been adapted to help manage a wide variety of acute and chronic pain conditions [2,3]. With rapid advancements in the field, effective doctor–patient communication is vital to ensure patients are made aware of all available evidence-based treatments, ensure they are able to openly discuss treatment preferences, and ensure they can reach an agreement with their provider regarding the most suitable treatment options [4]. This process is referred to as Shared Decision Making (SDM) and has been applied in a wide variety of disciplines [5,6,7]. Several studies have shown that greater patient involvement in care-related decisions leads to a higher level of patient satisfaction and the use of decision aids can help improve patients’ understanding of their clinical condition and available therapies [8,9].

Shared Decision Making has also been utilized in a variety of pain conditions including musculoskeletal pain, cancer pain, and palliative medicine patients [10,11,12]. SDM was found to be associated with a higher rate of patient satisfaction with chosen analgesic treatment among elderly patients presenting to the emergency medicine department with acute musculoskeletal pain [13]. The fundamentals of SDM when applied correctly can foster a trusting relationship between the patient and provider; however, its effects on clinical outcomes remain unclear. In patients with non-chronic, lower back pain, SDM did not result in improved either short-term or long-term recovery [14]. While SDM can help reaffirm the importance of selecting a treatment in line with patient preferences and can bolster patient engagement, it remains unclear if and how this practice translates to improved or quicker recovery.

When treating pain, it can be challenging to develop and sustain a therapeutic atmosphere as there may be disagreements regarding pain etiology. In addition, the use of opioids, a mainstay of pain treatment, has become controversial considering the ongoing opioid epidemic. Efforts to curb this national crisis via the implementation of prescription drug monitoring programs have shown reductions in the national opioid prescribing rate of 20.7% and 22.8% among oncologists and non-oncologists, respectively, between the years 2013 and 2017 [15]. Some patients suffering from pain view opioids as the strongest available treatment [16]. As such, a provider’s reluctance to prescribe opioids can create disagreements about available treatment options resulting in a strained doctor–patient relationship. The above-mentioned challenges are compounded when treating pain in patients with a history of opioid use disorder (OUD). OUD is characterized by the compulsive and continuous use of opioids despite negative medical, emotional, and social consequences [17]. Medical management of OUD involves methadone, buprenorphine, or naltrexone which are a mu-opioid receptor full agonist, partial agonist, and antagonist, respectively. Patients receiving these types of medications for the treatment of OUD (MOUD) may physiologically require higher opioid dosages for pain relief due to the variable changes made to receptor sensitivity and tolerance [18]. These overriding effects on the opioid receptor may make it difficult for providers to differentiate drug-seeking behaviors from patients who are undermedicated [19]. 

Furthermore, patients with OUD being treated for acute pain may have several worries including fears of withdrawal, relapse into unrestricted opioid use, feeling their pain may be undertreated, or fears of discrimination based upon prior substance use history. Physicians treating patients with OUD, especially those not familiar with MOUD, may also have concerns about the fabrication of pain, risk of opioid diversion, or overtreatment resulting in opioid-induced respiratory depression [20]. In such cases, SDM can act as a valuable tool to encourage open communication between patients and providers as well as to help ensure that the opinions and values of both parties are taken into consideration. However, information regarding the use of SDM for treating acute pain in patients with a history of OUD remains scarce. This scoping review aims to further describe the use of SDM in this patient population, observed effects on clinical management, and specific challenges that may be encountered in its implementation. 

## 2. Materials and Methods

### 2.1. Design

Compared to systematic reviews, scoping reviews are used to identify gaps in knowledge and examine research questions related to a particular theme. The protocol for this study has not previously been published. The research topic was explored with a medical librarian to develop the search strategy across multiple databases, and the Preferred Reporting Items for Systematic Reviews and the Meta-analysis Extension for Scoping Reviews (PRISMA-ScR) checklist was used to record the workflow (Supplementary Materials File S1) [21]. 

### 2.2. Identification of the Research Question

Research question: how is shared decision making used for pain management within the patient population with opioid use disorder? 

### 2.3. Search Strategy

The initial research strategy was developed for Ovid MEDLINE and then translated to PsychInfo, Embase, and CINAHL. The selection of search terms was iterative and included the summation of three search categories: “opioid use disorder”, “acute pain”, and “shared-decision making”. A combination of MeSH, EMTREE/Subject Headings, and keywords were included. The full search strategy for Ovid MEDLINE can be found in Supplementary Materials File S2. The search occurred in October 2022 and there were no time limits or language restrictions. The resulting records from all searches were merged; duplicates were deleted; and titles and abstracts were reviewed for eligibility. 

### 2.4. Eligibility Criteria

Articles were included if they involved patients with OUD or substance use disorder (SUD), discussed pain management in an acute setting, were peer-reviewed, and were original research, reviews, or reviews of clinical guidelines. Articles were excluded if they focused on the prevention of first-time OUD treatment, chronic pain, palliative care, or were oncology related. ASC and AM screened the initial list of records. Eligible abstracts were then reviewed for full-text and those that, upon further review, did not meet the criteria were excluded. Records where there was agreement to include or exclude were kept in their respective assignment; for records where there was disagreement or uncertainty based on the title/abstract, the articles were kept in the search. When there was a disagreement in final eligibility, a third author (TCL) handled the dispute. 

### 2.5. Charting the Data

The final set of articles was reviewed, charted, and the study details were extracted. Three authors (VB, AM, and PV) extracted article details including authors on the publication, year of publication, article type, population, treatment setting, and relevant findings. ASC grouped the articles by subtopics and the groupings were validated by the initial reviewers (VB, AM, and PV). Articles were grouped into subtopics based on the components of SDM described by the 1997 model developed by Charles et al. [22]. 

## 3. Results

### 3.1. Characteristics of Included Findings

**Figure 1:**
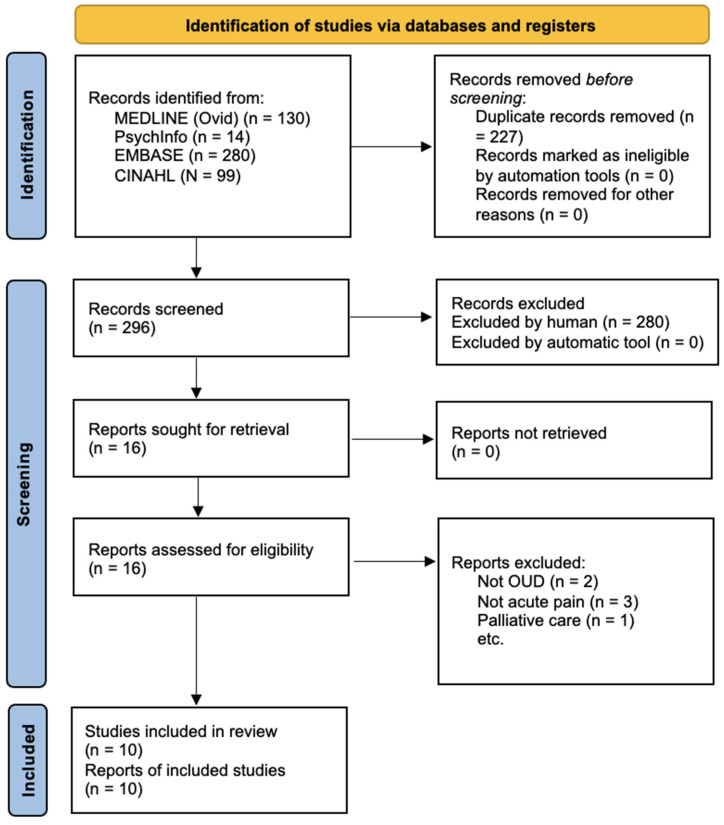
PRISMA Diagram.

The initial search retrieved 523 articles. Of these, 130 were found through Medline, 14 through PsychInfo, 99 through CINAHL, and 280 through EMBASE. After removing duplicates, 296 articles were reviewed. A total of 2 articles were excluded that were animal studies, 12 that did not focus on pain or pain management, 258 that did not focus on the correct population, and 8 records that were abstracts, dissertations, and educational material. A full review was performed on the remaining 16 articles. The complete search strategy is shown in Figure 1. Six records were excluded as they focused on the following: opioid dependency (n = 1), pregnancy but not acute pain (n = 2), chronic pain (n = 1), palliative care (n = 1), and intoxication (n = 1). The remaining 10 articles were included in the analysis. 

### 3.2. Study Findings

There were four (40%) original research/work articles, three (30%) reviews, and three (30%) clinical guidelines. Of the original research/work studies, one was a quality improvement intervention, two were surveys (one of patients and one of healthcare providers), and one was a case study. Two of the studies focused specifically on acute pain in the setting of endocarditis in patients with active intravenous drug use. Of the reviews and clinical guidelines, three were focused on patients receiving MOUD, two were focused on pain in patients with a history of SUD, and the last focused on pain management in patients with OUD in the critical care setting. Four themes emerged from the review: prior judgment and stigma related to OUD, trust and sharing of information, clinical tools, and interprofessional teams. A detailed summary of the results is presented in Table 1.

#### 3.2.1. Prior Judgment and Existing Stigma Related to OUD

Both the Butt et al. 2022 study and the review of clinical guidelines by Mefford and Donaldson discuss how prior experiences and existing stigma hinder SDM in pain management in patients with OUD [25,28]. Stumbo et al., 2017 reflected on how patients may stigmatize themselves [31]. Healthcare workers reported having an automatic bias before treating a patient with OUD and did not expect a positive outcome, and other providers stated they felt patients may be a “lost cause” due to their condition [25]. Some patients saw themselves as “addicted to medicine” not to illicit drugs; furthermore, they felt receiving treatment in a clinic with other individuals with OUD was “demoralizing” [31]. Some healthcare providers had unrealistic expectations regarding recovery, the ability to source housing, or to maintain abstinence from opioids which increased a negative bias toward the patient. The most extreme result was a refusal to perform repeat surgery when patients relapsed and suffered from endocarditis a second time [25]. Patients may also risk abrupt cessation of MOUD when admitted to the hospital for acute or emergency episodes, as some providers may fear adverse effects or may misconstrue symptoms of pain as drug-seeking behavior [28]. However, provider education on the pharmacologic properties of various medications taken by this population and working with an interdisciplinary team consisting of social workers, therapists, and pharmacists can allay some of these misconceptions [28]. 

#### 3.2.2. Trust and Sharing of Information 

In addition to education and a general understanding of the population, trust and the patient–physician relationship are essential for SDM. This theme is illustrated in several ways throughout the literature with an overlap across articles. First, Stumbo et al. illustrate the importance of patients sharing their history of substance use and how providing discrete details identifies patient perceptions relevant to treatment barriers and suitable options for controlling pain [31]. Second, healthcare workers may underestimate a patient’s level of pain or not believe a patient’s pain self-assessment score [27,28,29]. Third, a key responsibility of healthcare staff is to provide the education and empowerment tools to patients necessary for decision making [20,24,27]. Often, when patients are suffering from severe OUD or are having withdrawal symptoms, they are not making their best decisions; the role of the physician nevertheless remains to treat the patient with autonomy and provide the available options [30].

#### 3.2.3. Clinical Tools Such as Decision Aids, Guides, and Treatment Plans

There are several clinical tools with potential benefits in SDM. Broughton-Miller and Urquhart published the results of their institution’s quality improvement (QI) initiative for trauma patients on MOUD [23]. Their QI project was composed of four parts including a risk assessment, a shared decision-making tool, a provider’s checklist, and team engagement. The authors describe several considerations when employing a new tool. For example, their initial risk assessment was only administered by providers; however, later in the plan-do-study-act cycle, they added the ability for nursing to complete the assessment [23]. This addition resulted in more patients reporting pain scores less than 5 (from 20% to 78%), decreased average pain scores from 8 to 4.6, and reported patient engagement with SDM to 92%. Krashin et al. also discuss the importance of risk assessment and mention several existing tools such as the Current Opioid Misuse Measure (COMM) (with a sensitivity of 77% and a specificity of 77% for identifying patients with OUD), the Opioid Risk Tool (specificity of 85.1% and sensitivity of 85.4%), and the Screener and the Opioid Assessment for Patients with Pain (SOAPP) (a sensitivity of 75% and specificity of 80% in identifying OUD) [20]. Furthermore, Mitchell et al. discuss a “Hospital Misuse Checklist” administered by addiction psychiatry to monitor addictive behaviors [29]. In addition to a risk assessment tool, Broughton-Miller and Urquhart’s study also utilized a modified Ottawa Personal Decision guide which has improved patient knowledge, increased accurate risk perceptions, and increased congruence between informed values and chosen options [23]. Utilizing such a tool is in line with the Agency for Healthcare Research and Quality (AHRQ) SHARE Approach [32]. Finally, Broughton-Miller and Urquhart describe a third clinical tool, a provider’s checklist which they created [23]. This checklist led to more accurate care including the resumption of MOUD treatment. 

#### 3.2.4. Interprofessional Teams

The last subtopic of SDM explored in this scoping review was interprofessional care between providers and healthcare workers. This was a recurrent theme across several articles. Collaboration during the acute pain episode between the attending doctor and clinical care team (physicians, nurse practitioners, and social workers) led to increased communication with the patient in the Broughton-Miller and Urquahart study [23]. Mefford and Donaldson recommended increased collaboration between pharmacists and the medical team in order to provide education and assist with the pharmacology [28]. Smith et al. discuss the addition of addiction, mental health, and pain specialists [30]. Many of the articles discuss the importance of discharge planning coordination with various providers including social workers, discharge planning staff, outpatient pain clinicians, and outpatient providers including the providers prescribing MOUD [24,26]. Although many of the articles discussed the importance of interprofessional teams, only Broughton-Miller and Urquhart discuss the logistics; in their study the teams had 1-min huddles [23]. In the busy hospital environment where addiction medicine may be seated in another wing and/or the outpatient MOUD and provider does not utilize an electronic health record system, communication may be harder. Our review shows that having communication and interprofessional teamwork optimizes patient care and shared decision making but how to make that routinely occur may still be unclear. 

## 4. Discussion

This scoping review consolidated and expounded the current literature on SDM in the management of acute pain in patients with OUD, the observed effects on clinical management, and specific challenges that providers may face in its execution. Our review found the various components of SDM, as described by Charles et al., across the 10 articles [22]. The themes were well distributed and ranged from previous experience and stigma, limitations of trust and information, clinical utility, and interprofessional teams. 

The discussion of prior judgment and existing stigma related to OUD is well established in the initial decision of a provider to prescribe opioids. Some of the barriers related to this component of SDM include generalized and blanketed approaches, uninformed consent, fear of opioids or pain, mental health disorders, lack of agency, lack of choice, and increased stigma regarding addiction. These barriers appear to be common across multiple specialties, including primary care, telemedicine, perinatal, postpartum, and cancer management [33,34,35,36,37]. Additionally, high levels of stigma envelop OUD and are pervasive throughout the entirety of the healthcare system, from early years of the school curriculum to independent practice. The lack of OUD and MOUD education, unrealistic provider expectations, and development of punitive policies towards OUD can result in lower interest from providers to work with OUD patients. Stigma and provider education are also barriers in other fields of medicine such as medical treatment including hormone therapy for those who identify as transgender [38,39]. Education and experience are a subcomponent in the building of trust and sharing of information which in turn allows for improved shared decision making. 

Trust and information sharing is a key component in shared decision making. A study by Ritter et al. showed that a 2-h training plus the use of an SDM protocol discussing patient efficacy, provider’s experience, and patient’s values increased standardized patients’ comfort with the discussion [40]. In part, due to the stigma mentioned previously, this level of comfort and trust within the physician–patient dyad may be more challenging to achieve in patients with OUD [41]. As mentioned in the study by Stumbo et al., patients may be less willing to disclose their substance use history [31]. At the same time, healthcare providers may struggle to understand and trust a patient’s self-reported pain score [20,27]. Coronado-Vázquez et al. discuss the benefits of using a decision aid in the process of SDM in the primary care setting [42]. Their systematic review included various SDM topics including selecting treatment for depression and selecting treatment for lower back pain. In their study, the authors found increased satisfaction from the patients and increased learning through the use of decision aids [42]. 

Research has shown success in the use of decision aids or clinical tools to support SDM. A study by Aarts et al. on women with heavy menstrual bleeding and/or uterine fibroids showed that training of clinicians and the use of the “Option Grid encounter” decision aids yielded higher SDM scores based on the patient self-reported collaboRATE 3-question measure [43]. A meta-analysis by Niburski et al. of SDM aids in the discipline of surgery showed overall decreased conflict, regret, and anxiety around the decision and increased knowledge and satisfaction [44]. Decision aids have also been used in the OUD population as a way to engage patients in recovery. Guille et al. studied the use of a decision aid to help pregnant women with OUD decide on a course of action for their MOUD therapy [45]. Participants reported that the SDM process with the use of the decision aid provided sufficient information and that they were able to make decisions in line with their values. Another OUD treatment-based study by Mooney et al. found that their decision tool around the decision to start MOUD, based on the Patient Decision Aid Standards, resulted in more participants both starting therapy and staying in therapy [46]. In our review, we found one study, a quality improvement initiative, that utilized a decision aid, namely a modified Ottawa Personal Decision Tool [6]. Although this was the only decision-aid tool we found, other clinical tools were mentioned throughout the literature including risk assessments, opioid misuse scores, and provider checklists [20]. As Niburski et al. mention in their meta-analysis, there is insufficient information in the literature as to which decision aids and tools in SDM are optimal for the said decision and population [44].

Although uncertainty exists with respect to the type of decision aids to use and is an area of future research, one area of shared decision making that appears consistent across the literature is the use of a multidisciplinary team. This aspect of SDM reoccurred frequently in our review. In the area of critical care, Smith et al. discuss the need for regular patient monitoring and for communication with the patient when possible [30]. Krashin et al. note that patients prefer a frank and direct conversation [20]. To ease these conversations and others, the inclusion of other healthcare professionals may be beneficial. Pharmacists may be able to help providers understand the pharmacology of MOUD and provide guidance on how best to manage the medication, thereby easing provider concerns and possibly allowing them to listen to the patient’s needs without so much fear [28,47]. Butt et al. discuss the role of social workers and Hickey et al. discuss the roles of both inpatient and outpatient clinicians and discharge coordinators [25,26]. Buresh et al. discuss consulting with the patient’s outside MOUD provider and many of the articles we reviewed discussed the inclusion of addiction specialists [20,24,26]. The inclusion of these professionals assists with providing the patient with the education they need in order to make a value-directed decision. With team engagement, professionals are better able to communicate with each other and the patient and work towards the goal of helping the patient [23]. With a shared goal and support from others, it may be easier to set aside their prior judgments. 

The use of a multidisciplinary team in shared decision making has been seen in other areas of medicine. A 2018 publication by Quinn et al. discusses their team’s implementation of a multidisciplinary decision-making process among clinicians followed by shared decision making with the patient/caregiver in the pre-operative oncology setting [48]. Nanapragasam et al. propose a different multidisciplinary shared decision-making approach where the patient is included in the multidisciplinary discussion between orthopedic, radiologist, and other providers in the case of back pain [49]. These successful implementations of shared decision making in a multidisciplinary team highlight the feasibility and benefits of such a process.

In the setting of acute pain management for patients with OUD or with a history of OUD, there is the opportunity to do the same. SDM has the potential to be a valuable skill. Our research shows that although some work has been conducted in this field, more is necessary. Increased efforts are needed to continue to reduce stigma, increase provider and patient education, and build trust [41]. Clinical tools such as risk assessments and decision aids must be assessed while continuing to work as a team. A 2015 news report from the National Institute of Health estimated that roughly 10% of US adults either have or have had a substance use problem in their lifetime [50]. In response to this, legislation has been passed which allows the purchase of naloxone without a prescription as well as prescriptions written to third-party family members of patients who are on opioid therapies. Efforts continue to be made by opioid overdose education and community naloxone distribution programs, though further research is needed to investigate the best means of outreach in order to educate the public on the signs and symptoms of opioid overdose as well as the appropriate administration of naloxone when an overdose is suspected [51]. Given the prevalence of substance use disorders and overdose-related deaths, it is vital that we develop a patient-centered process to address acute pain in this population. 

### 4.1. Limitations

Our scoping review has several limitations. Although our focus was on OUD, there were articles within our search on SUD that we opted to include although not in our original question. Although SUD includes OUD, these articles may have been referencing situations not related specifically to OUD. Additionally, unlike a traditional scoping review, we had a framework around SDM that we were attempting to address through the literature. This may have prevented us from analyzing the data in a different way, possibly leading to other factors that may be significant in SDM in this population. Finally, the inclusion of reviews of clinical guidelines in a scoping review is atypical. Our decision to include these articles was based on (1) the limited literature we expected to find and (2) a route to understanding how clinical guidelines impact providers in the SDM process.

### 4.2. Strengths

Despite our limitations, to our knowledge we offer the first scoping review that explores the relationship between SDM and the management of acute pain in patients with OUD. Using several databases, we were able to pull data relating to physicians in various settings as well as nurses, pharmacists, and other healthcare professionals. We were also able to explore the various components that impact SDM including the individual, clinical guidance, and actual execution of the process.

### 4.3. Implications for Practice

There is an insidious stigma that goes beyond patients and extends to providers with direct patient care, influencing systemic utilities of pharmacies, insurance, housing, and transportation, among others, which creates further barriers for patients with OUD. This creates an additional barrier for those who are actively seeking treatment for OUD. Clinical utilities used during clinical visits have shown usefulness in SDM. Using a risk assessment, a shared decision-making tool, a provider’s checklist, and team engagement have all established guidelines to diagnose, treat, and monitor OUD. Without these tools, meticulous management and record-keeping would not be plausible, leaving room for errors.

## 5. Conclusions

Increased patient participation is always desired during treatment. Collaboration between the attending doctor and the clinical care team leads to increased communication with the patient, promoting care, and understanding among all parties. Increased pharmacist–physician communication also provides clear education and increases treatment adherence for patients and physicians. Having dedicated addiction, mental health, and pain specialists increases resources and decreases barriers to accessing treatment for patients with OUD. More importantly, access to discharge planning coordination with various providers, including social workers, discharge planning staff, outpatient pain clinicians, and outpatient providers will improve treatment outcomes.

## Figures and Tables

**Table 1:** Table of articles included in the analysis of Shared Decision Making and patients with Opioid Use Disorder.

Study	Type of Article	Patient Population	Outcomes and Findings	Subtopic
Broughton-Miller and Urquhart, 2022 [23]	Original Research	Inpatient trauma patients with OUD and on MOUD	Modified Ottawa Personal Decision tool led to high levels of patient engagement Improved pain outcomes with four-pronged intervention: risk assessment, provider checklist, and healthcare team engagement	CT, MT
Buresh et al., 2020 [24]	Review	Men and women on buprenorphine undergoing surgery	Continue buprenorphine and use a multimodal pain management approach Close communication with the patient’s outpatient MOUD provider General mention of shared decision making and individualized care but no detailed recommendations	TS, MT
Butt, et al., 2022 [25]	Original research	IV drug use and infective endocarditis	Healthcare workers’ stigma led to barriers in care Need for improved communication SDM needs to also include post-hospital substance use treatment	JM
Hickey et al., 2022 [26]	Review of clinical guidelines	OUD patients on buprenorphine undergoing surgery	Multidisciplinary approach Mention of patient-centered care but no mention of shared decision making between patient and provider	MT
Krashin et al., 2012 [20]	Review	Acute and chronic pain patients with a history of SUD	Frank and open discussions regarding pain management and planning Patient education and treatment agreements Universal risk assessment Motivational interviewing for patients active in SUD	TS, CT
Kurtz, 2003 [27]	Review of clinical guidelines	Acute pain patients with a history of SUD	Improve patient education and empowerment Active discussion of available therapies Coordination of treatment goals between patients and providers	TS, MT
Mefford and Donaldson, 2022 [28]	Review of clinical guidelines	OUD patients on MOUD with acute pain	Barrier: Provider perceptions of OUD patients Barrier: Provider lack of knowledge on MOUD Mention of including patient preferences in the management of MOUD and pain	JM, TS
Mitchell et al., 2020 [29]	Original research	Endocarditis associated with IV drug use	Involve addiction specialists Nursing education Tools to identify aberrant behavior may be useful Acute pain an opportunity for SDM in recovery engagement	TS, CT, MT
Smith et al., 2022 [30]	Review	ICU/perioperative OUD patients	Pain scales and monitoring for withdrawal symptoms Multidisciplinary team and discharge planning	TS, CT, MT
Stumbo et al., 2017 [31]	Original research	Pathways to OUD and pain barriers	Prescription of opioids may result in recurrence even after years of abstinence Patients with previous substance use may not disclose their history	JM, TS

OUD = opioid use disorder; MOUD = medications for opioid use disorder; IV = intravenous; SDM = shared decision making; SUD = substance use disorder; ICU = intensive care unit. JM = Prior judgment and existing stigma related to OUD; TS = Trust and sharing of information (both ways); CT = Clinical tools such as decision aids, guides, and treatment plans; MT = Provider collaboration, communication between healthcare workers, and multidisciplinary team.

## Data Availability

No new data were created or analyzed in this study. Data sharing is not applicable to this article.

## Supplementary Materials

The following supporting information can be downloaded at: https://www.mdpi.com/article/10.3390/jcm12103555/s1. Supplementary Materials File S1: Preferred Reporting Items for Systematic Reviews and Meta-Analyses extension for Scoping Reviews (PRISMA-ScR) checklist; Supplementary Materials File S2: MEDLINE (Ovid) Search Strategy.
