# Supplementary material for: Shared Decision Making in Acute Pain Management in Patients with Opioid Use Disorder: A Scoping Review
Source: J Clin Med. 2023 May 19;12(10):3555. doi: 10.3390/jcm12103555 (PMC10219029; doi:10.3390/jcm12103555)
Supplement: Supplementary file 1 [file jcm-12-03555-s001.zip › Supplementary Materials File S2.pdf]

Supplementary Materials File S2. MEDLINE (Ovid) Search Strategy.

1 ((opioid* or fentanyl) adj3 (abus* or use* or disorder*)).mp.

2 ((acute or surg* or perioperat* or operat* or postoperat* or trauma or peripartum or postpartum) adj3 pain).ab,kf,kw,ti.

3 Acute Pain/

4 decision making/ or decision making, shared/

5 Patient Participation/

6 (patient* adj3 (empower* or engage* or involv* or participat*)).mp.

7 (decision* adj2 (made or make or making)).mp.

8 "shared-decision".mp.

9 Opioid-Related Disorders/

10 1 or 9

11 4 or 5 or 6 or 7 or 8

12 2 or 3

13 10 and 11 and 12
